# Influence of Ectopic Beats on Heart Rate Variability Analysis

**DOI:** 10.3390/e23060648

**Published:** 2021-05-22

**Authors:** Lina Zhao, Peng Li, Jianqing Li, Chengyu Liu

**Affiliations:** 1 The State Key Laboratory of Bioelectronics, School of Instrument Science and Engineering, Southeast University, Nanjing 210096, China; 101102013@seu.edu.cn; 2 Division of Sleep and Circadian Disorders, Brigham and Women’s Hospital, Harvard Medical School, Boston, MA 02115, USA; pli9@bwh.harvard.edu

**Keywords:** electrocardiogram, sample entropy, congestive heart failure, heart rate variability

## Abstract

The analysis of heart rate variability (HRV) plays a dominant role in the study of physiological signal variability. HRV reflects the information of the adjustment of sympathetic and parasympathetic nerves on the cardiovascular system and, thus, is widely used to evaluate the functional status of the cardiovascular system. Ectopic beats may affect the analysis of HRV. However, the quantitative relationship between the burden of ectopic beats and HRV indices, including entropy measures, has not yet been investigated in depth. In this work, we analyzed the effects of different numbers of ectopic beats on several widely accepted HRV parameters in time-domain (SDNN), frequency-domain (LF/HF), as well as non-linear features (SampEn and Pt-SampEn (physical threshold-based SampEn)). The results showed that all four indices were influenced by ectopic beats, and the degree of influence was roughly increased with the increase of the number of ectopic beats. Ectopic beats had the greatest impact on the frequency domain index LF/HF, whereas the Pt-SampEn was minimally accepted by ectopic beats. These results also indicated that, compared with the other three indices, Pt-SampEn had better robustness for ectopic beats.

## 1. Introduction

The concept of heart rate variability (HRV), the tiny fluctuations in beat-to-beat heart rate, was first proposed by Hon and Lee in 1965 [1]. It is an important index to evaluate the functional status of the cardiovascular system, as well as the various physiological and clinical conditions, reflecting the ability of cardiovascular autonomic regulation [2]. HRV is based on the analysis of RR intervals from the beats generated by the sinoatrial node and can be analyzed in many different ways, including time- and frequency-domain analyses and non-linear analyses [3]. HRV can be used in the evaluations of different cardiovascular diseases, such as arrhythmia [4], acute myocardial infarction [5], coronary heart disease [6], and hypertension [7]. However, bias can be introduced in HRV analysis due to artifacts. A typical example of biological artifacts is ectopic beat [8], which is not generated by the sinoatrial node and has shown to significantly bias the analysis of HRV [9,10,11].

The Task Force of the European Society of Cardiology and the North American Society of Pacing Electrophysiology recommended interpolation methods such as linear regression or similar algorithms on editing ectopic beats or on post-processing its autocorrelation function may decrease this error [4]. Exclusion of ectopic segments of RR interval time series from the analysis has been used, but this technique may still bias HRV results if ectopic beats are causally associated with changes in autonomic tone. Lippman et al. assessed three methods (deletion, linear and cubic spline interpolation, non-linear predictive interpolation) for correcting for ectopy and found deletion and non-linear predictive interpolation performed superiorly to linear or cubic spline interpolation, which overestimated or underestimated frequency-domain HRV indices [12]. Wen and He proposed a method for predicting the RR interval value at an ectopic beat time based on weight calculation and slope estimation of preceding normal RR intervals and demonstrated the efficiency on power spectrum density (PSD) estimation [13]. Mateo and Laguna also developed a method for dealing with the PSD estimation of the HRV by means of the heart timing signal when ectopic beats are present [14]. Although many methods for detecting and editing ectopic beats have been proposed, there is not a widely agreed conclusion on how to efficiently handle them to obtain accurate HRV indices. The lack of such a guideline may partially be due to different sensitivities of HRV indices to the burden of ectopic beats [15,16]. For example, Ahyoung and Hangsik quantitatively analyzed the effect of ectopic beats on HRV indices in resting condition, and their results showed that the traditional time-domain and frequency-domain indices obtained after ectopic beats interpolation differed significantly from those prior to the interpolation even when the portion of ectopic beats is low [17].

Unlike traditional time-domain and frequency-domain HRV indices, entropy is a newly developed non-linear measure in the past thirty years that exhibits good performance on short-term physiological signal analysis [18,19]. Entropy is a valuable tool for quantifying the complexity or irregularity of cardiovascular time series [20]. There are various algorithms established for performing the entropy analysis, such as approximate entropy (ApEn) and sample entropy (SampEn). The SampEn has been proved more stable and consistent statistically than ApEn since SampEn excludes the self-matching in its calculation [21,22], and there are also various improvement methods based on ApEn and SampEn [23,24]. Ectopic beats can contaminate the entropy calculations as well [17,25]. The quantitative relationship between the burden of ectopic beats and HRV indices, including entropy measures, has not yet been investigated in depth.

Therefore, in this work, we aimed to examine the effect of the burden of ectopic beats on different HRV indices, including traditional time- and frequency-domain measures as well as entropy measures, and to compare these influences between patients with congestive heart failure (CHF) and normal sinus rhythm (NSR) subjects.

## 2. Methods

### 2.1. Data and Preprocessing

The data used in this study were from the free-access PhysioNet/MIT-BIH RR Interval Databases (http://www.physionet.org, accessed on 1 September 2015) [26]. There were two RR interval databases: the NSR RR Interval Database (54 subjects with normal sinus rhythms and aged from 29 to 76 years), and the CHF RR Interval Database (29 subjects aged from 34 to 79 years, with New York Heart Association (NYHA) CHF diagnose classes I, II, and III). Each subject had a long-term RR interval recording of nearly 24 h, which was from an electrocardiogram (ECG) recorded under a similar level of physical activity using a Holter monitor (digitized at a rate of 128 Hz). Beat annotations were obtained by automated analysis with manual review and correction. The long-term RR interval records were cut into 5 min segments without overlap. The 5 min RR segments with at least one ectopic beat were extracted as ectopic segments in this study. Table 1 and Table 2 summarize the numbers of ectopic-free and ectopic 5 min segments in separate NSR and CHF groups from the PhysioNet/MIT RR Interval Database.

**Figure 1:**
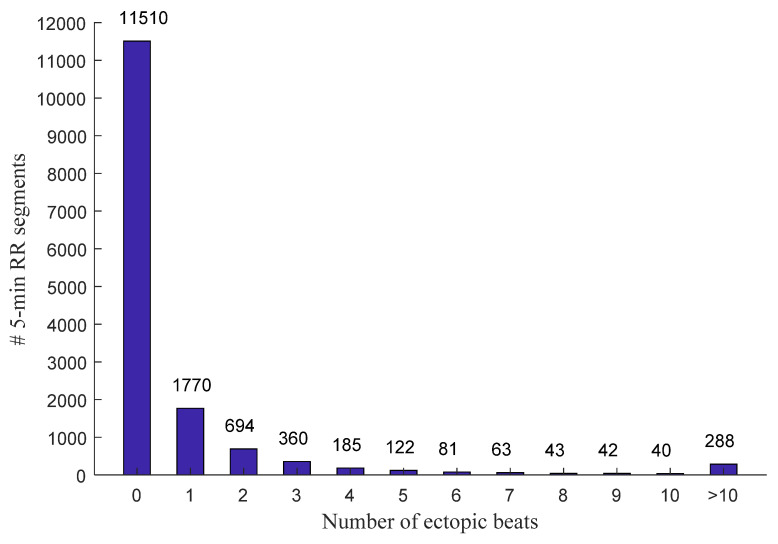
Distribution of the ectopic-free and ectopic 5 min RR segments classified by the number of ectopic beats in the NSR group. # Number of.

**Figure 2:**
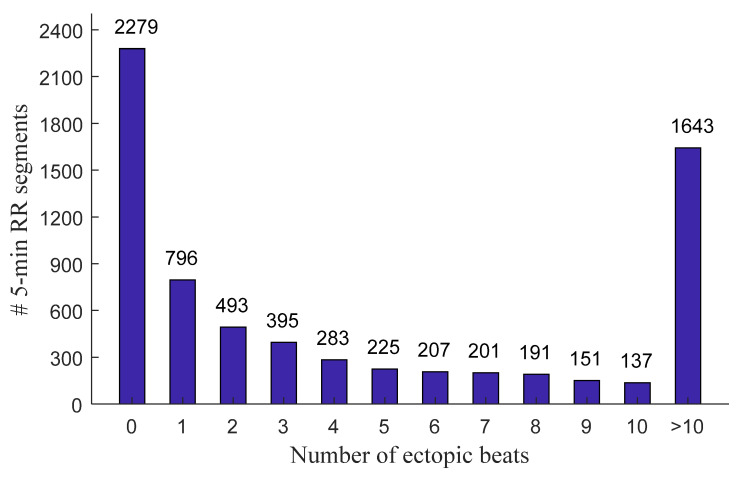
Distribution of the ectopic-free and ectopic 5 min RR segments classified by the number of ectopic beats in the CHF group. # Number of.

Figure 1 and Figure 2 show the distribution of the ectopic-free and ectopic 5 min RR segments classified by the number of ectopic beats in both NSR and CHF groups. In the NSR group, there were 11,510 ectopic-free segments in total, and 1770, 694, 360, 185, 122, 81, 63, 43, 42, and 40 ectopic segments with an ectopic beats increase from one to ten, respectively. In addition, there were 288 ectopic segments with ectopic beats >10. In the CHF group, there were 2279 ectopic-free segments in total, and 796, 493, 395, 283, 225, 207, 201, 191, 151, and 137 ectopic segments with an ectopic beats increase from one to ten, respectively. Meanwhile, there were 1643 ectopic segments with ectopic beats >10.

**Figure 3:**
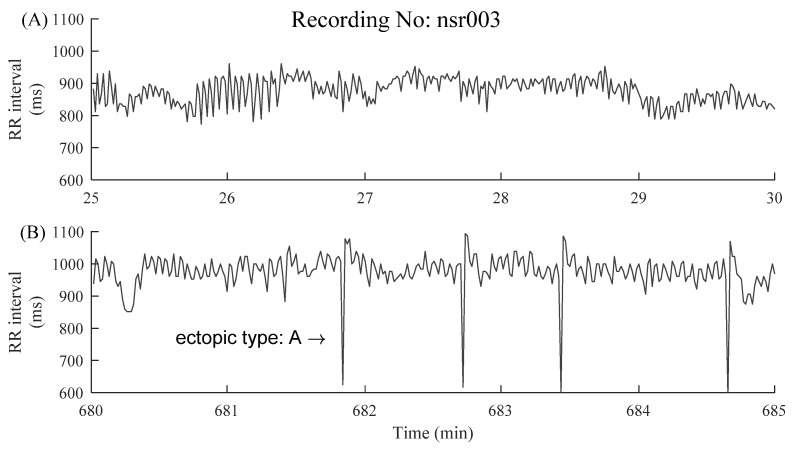
Examples of 5 min RR segments from an NSR subject: (**A**) 5 min RR segment without ectopic beats and (**B**) 5 min RR segment with premature atrial contraction (ectopic type A). The x-axes show the real-time window from the raw RR interval recordings, facilitating the readers to locate these selected 5 min RR segments.

**Figure 4:**
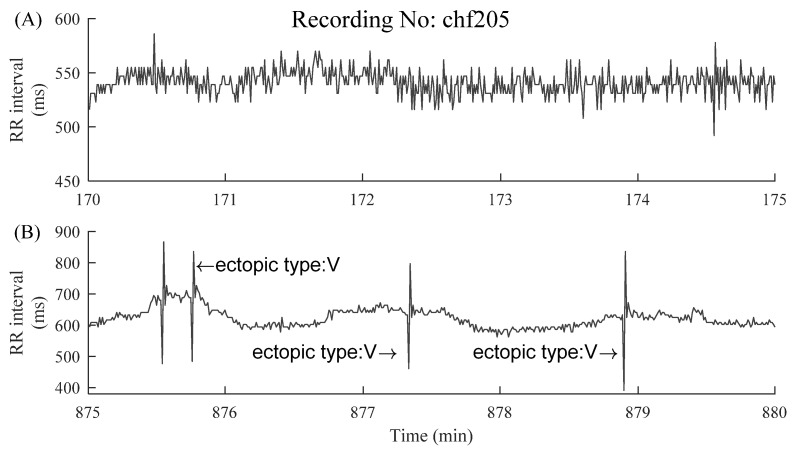
Examples of 5 min RR segments from a CHF patient: (**A**) 5 min RR segment without ectopic beats and (**B**) 5 min RR segment with premature ventricular contraction (ectopic type: V). The x-axes show the real-time window from the raw RR interval recordings, facilitating the readers to locate these selected 5 min RR segments.

The information regarding ectopic beats was manually annotated by experts, which were classified as atrial (A) or ventricular (V) beats, depending on the localization of the ectopic focus. In each segment, the RR intervals greater than 2 s, but not associated with ectopic beats, were removed in order to alleviate the potential influence of noises [27]. Figure 3 and Figure 4 show examples of ectopic-free and ectopic RR segments from the two groups. The length of each segment was fixed to 5 min.

### 2.2. HRV Indices

The commonly used HRV indices in the time-domain (i.e., SDNN), frequency-domain (i.e., LF/HF), and a non-linear index (i.e., SampEn) were studied in this study. In addition, in our previous study, a new physical threshold-based entropy (Pt-SampEn) for analyzing NSR and CHF groups was developed to improve the poor stability of the SampEn method [28]. Therefore, we also included the analysis of Pt-SampEn in this work as a comparison. We calculated the above-mentioned four indices for each of the 5 min RR segments.

1. SDNN: SDNN is the standard deviation of a 5 min RR segment, and was calculated as follows:
  (1) $$\mathrm{SDNN}=\sqrt{\frac{\sum_{i=1}^{N}{\left(RR_{i}-RR_{mean}\right)}^{2}}{N}}$$
  where $RR_{i}$ is the *i*th RR interval, $RR_{mean}$ is the mean value of all RR intervals, *N* is the number of RR intervals.
2. LF/HF: The frequency-domain features were calculated using power spectral density. Prior to the frequency-domain analysis, spline interpolation was used to resample the RR intervals time series evenly at 4 Hz. HRV spectrum was produced using Burg’s method with an order of 10 [24]. It was then decomposed into two separate frequency bands: a low-frequency band (LF, 0.04 to 0.15 Hz) and a high-frequency band (HF, 0.15 to 0.4 Hz). The ratio of low-frequency power to high-frequency power (LF/HF) was calculated, which reflected the balance between the sympathetic and parasympathetic (or vagal) activity.
3. Sample entropy: SampEn is a widely used index for HRV analysis and can reflect the inherent complexity or regularity of RR interval time series. The calculation for SampEn was summarized as follows [20]:
  For a 5 min RR segment $X=\left\{x_{1},x_{2},\cdots ,x_{N}\right\}$, given the parameters *m* and *r*, first form the vector sequences $X_{m}\left(i\right)$, which represented *m* consecutive $x_{i}$ values:
  $$X_{m}\left(i\right)=\left[x_{i},x_{i+1},\cdots ,x_{i+m-1}\right]\left(1\leq i\leq N-m\right).$$
  The distance between $X_{m}\left(i\right)$ and $X_{m}\left(j\right)$ was defined using the maximum absolute difference:
  $$d_{i,j}^{m}=d(X_{m}\left(i\right),X_{m}\left(j\right))=\max\left(\left|x_{i+k}-x_{j+k}\right|\right)\left(0\leq k\leq m-1\right).$$
  For each $X_{m}\left(i\right)$, denote $B_{i}^{m}\left(r\right)$ as (N-m)-1 times the number of $X_{m}\left(j\right)$ (1≤j≤N−m, j≠i) that meets $d_{i,j}^{m}\leq r$. Similarly, set $A_{i}^{m}\left(r\right)$ is ${\left(N-m\right)}^{-1}$ times the number of $X_{m+1}\left(j\right)$ that meets $d_{i,j}^{m+1}\leq r$ for all 1≤j≤N−m.
  Then SampEn is defined by:
  $$\mathrm{SampEn}\left(m,r,N\right)=-\ln\left(\sum_{i=1}^{N-m}A_{i}^{m}\left(r\right)/\sum_{i=1}^{N-m}B_{i}^{m}\left(r\right)\right).$$
  In the current study, the parameter settings for SampEn used the recommendation from our previous study [22], i.e., embedding dimension *m* = 2 and tolerance threshold *r* = 0.10 times the standard deviation (SD) of the time series.
4. Physical threshold-based SampEn (Pt-SampEn): For entropy analysis, three intrinsic parameters (embedding dimension 𝑚, tolerance threshold *r*, and time-series length *N*) needed to be initialized. SampEn was reported not sensitive to the time series length if N≥200~300 but sensitive to the parameter tolerance threshold. Since parameter *m* is based on the length *N* ($N\approx 10^{m}\sim 20^{m}$), SampEn was also not sensitive to *m*. Tolerance threshold *r* was difficult to be determined and was recommended between 0.10 and 0.25 times the SD of the time series. However, in practice, if the *r* value was too small, the number of matched vectors was small, and, on the contrary, if the *r* value was too large, detailed information within the time series was ignored. RR interval time series usually have variable SD values, and it is not easy to find an appropriate *r* value to achieve an optimal result. Hence, simply use the suggested range of 0.10 to 0.25 times the SD. Herein, a physical threshold *r* was used to form a unified comparison baseline for determining the vector similarity, and, thus, the Pt-SampEn was developed in our previous study [28].

As the raw ECG signals were sampled at 128 Hz indicating that the difference between any two vectors was approximately an integer multiple of 8 ms. Thus, we employed an *r* of 12 ms as the physical threshold [28]. In addition, previous studies suggested that using an *m* = 1 could obtain better results for classifying NSR and CHF groups with *N* = 300 [27]. In this study, we kept this suggested *m* = 1 for both SampEn and Pt-SampEn.

### 2.3. Evaluation Method

RR intervals associated with ectopic beats contaminate the calculations of HRV indices. Thus, these RR intervals should be removed from the original RR interval time series. For an atrial or ventricular ectopic beat annotation, the two RR intervals before and after this ectopic beat were removed. HRV indices that can better tolerate these abnormal intervals are thought to have more stability. In other words, the evaluation should rely on the relative change prior to and after the removal of intervals associated with ectopic beats. We thus defined the following change rate index C as a criterion to evaluate the performance of different HRV indices:

(2) $$C=\left|\frac{M_{after}-M_{before}}{M_{before}}\right|\times 100\%,$$

where $M_{before}$ and $M_{after}$ mean the values of the corresponding HRV index before and after removing ectopic beats.

**Figure 5:**
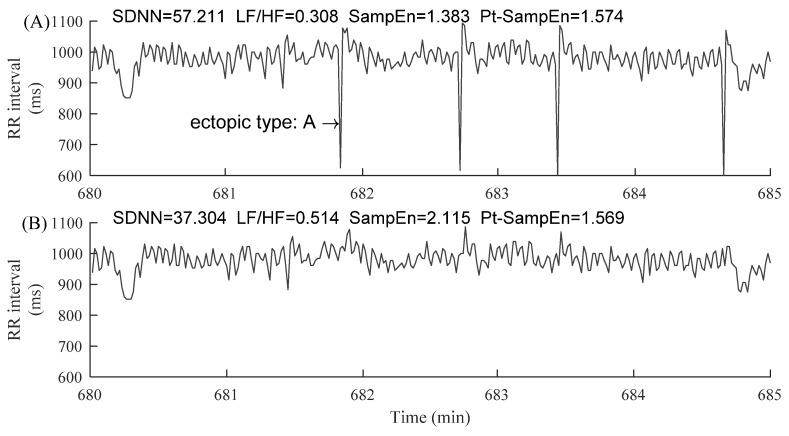
Demonstration of the changes of HRV indices before (**A**) and after (**B**) ectopic beats removal in a 5 min RR segment of subject NSR003. The corresponding HRV results were given in each sub-figure. “ectopic type: A” means premature atrial contraction.

**Figure 6:**
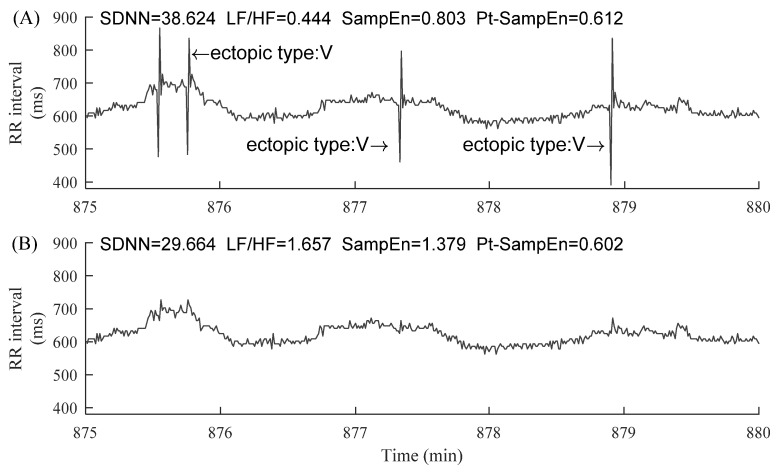
Demonstration of the changes of HRV indices before (**A**) and after (**B**) ectopic beats removal in a 5 min RR segment of patient CHF205. The corresponding HRV results were given in each sub-figure. “ectopic type: V” means premature ventricular contraction.

Figure 5 demonstrates the changes of HRV indices before and after ectopic beats removal in a 5 min RR segment of an NSR subject. After four atrial ectopic beats were removed, the results of index C were: $C_{\mathrm{SDNN}}=34.00\%$, $C_{\mathrm{LF}/\mathrm{HF}}=66.88\%$, $C_{\mathrm{SampEn}}=52.93\%$, and $C_{\mathrm{Pt}-\mathrm{SampEn}}=0.32\%$. Similarly, Figure 6 demonstrates the similar results from a CHF patient. After four ventricular ectopic beats were removed, the results of index C were: $C_{\mathrm{SDNN}}=23.20\%$, $C_{\mathrm{LF}/\mathrm{HF}}=273.20\%$, $C_{\mathrm{SampEn}}=71.73\%$, and $C_{\mathrm{Pt}-\mathrm{SampEn}}=1.64\%$. From these two typical examples, we could observe that the changes of indices of SDNN, LF/HF, and SampEn were much large after the ectopic beats removal, while the change of index of Pt-SampEn was relatively small.

### 2.4. Statistical Analysis

We classified the 5 min ectopic RR segments into 11 types based on the number of ectopic beats, i.e., ectopic beat number of 1, 2, 3, 4, 5, 6, 7, 8, 9, 10, and more than 10. To ensure the valid number of RR intervals in each 5 min RR segment, the RR segments with ectopic beat number > 50 were excluded for the following analysis. First, the four HRV index values for each 5 min ectopic RR segment were calculated separately for before and after ectopic beats removal. The HRV index results were tested as normal distribution by the Kolmogorov–Smirnov test, separately for each ectopic number group, as well as separately for the two groups. If the HRV index results met the normal distribution, the mean (SD) was reported to present the center and dispersion, and a paired *t*-test was used to test the statistical difference between before and after ectopic beats removal. If not, the median (interquartile range, IQR) was reported, and a non-parametric test was used. All statistical analyses were performed using the Statistical Package for Social Sciences (V19, SPSS Inc., Chicago, IL, USA), and a value of *p* < 0.05 was considered statistically significant. 

## 3. Results

All HRV index results (SDNN, LF/HF, SampEn, and Pt-SampEn) from both NSR and CHF groups did not meet the normal distribution of the Kolmogorov–Smirnov test. Thus, the median (IQR) results were reported. Significant statistical differences of HRV indices between before and after ectopic beats removal were observed, as we expected, for any ectopic number type as well as for both NSR and CHF groups. However, the new Pt-SampEn showed the least changes when removing the ectopic RR intervals from the original RR segment.

### 3.1. C_SDNN_

**Figure 7:**
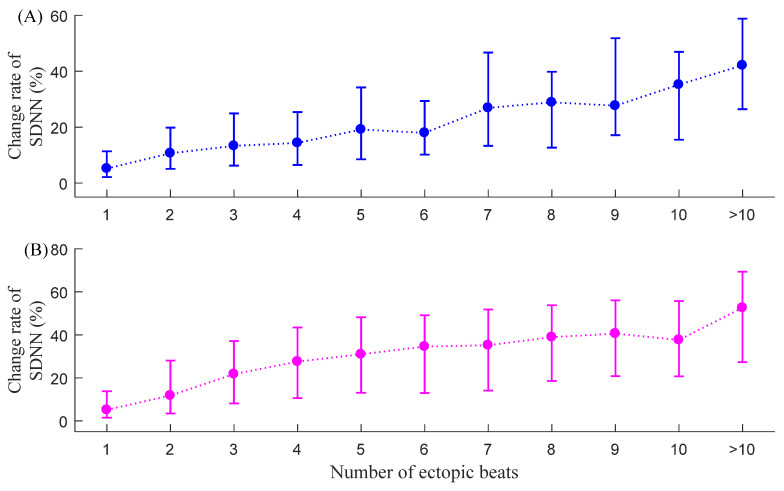
Trends of C_SDNN_ under different ectopic beat burdens, i.e., the number of ectopic beats increase from 1 to more than 10: (**A**) NSR group and (**B**) CHF group.

Figure 7 shows the corresponding median (IQR) results of C_SDNN_ for both NSR subjects and CHF patients. SDNN decreased as expected when the ectopic beats were removed, and its change rate index C_SDNN_ significantly increased with the increase of the number of ectopic beats. The increasing trend in the two groups was similar. For NSR subjects, C_SDNN_ results were 5.2% (2.2–11.4%) when only one ectopic beat was included in each 5 min RR segment and increased to 42.1% (26.4–58.8%) when more than 10 ectopic beats were included. For CHF patients, C_SDNN_ results were 5.2% (1.5–13.8%) when only one ectopic beat was included and increased to 52.6% (27.3–69.3%) when more than 10 ectopic beats were included. The C_SDNN_ results from the two groups indicated that the influence of ectopic beat burden for SDNN appeared to grow with the increasing number of ectopic beats.

### 3.2. C_LF/HF_

**Figure 8:**
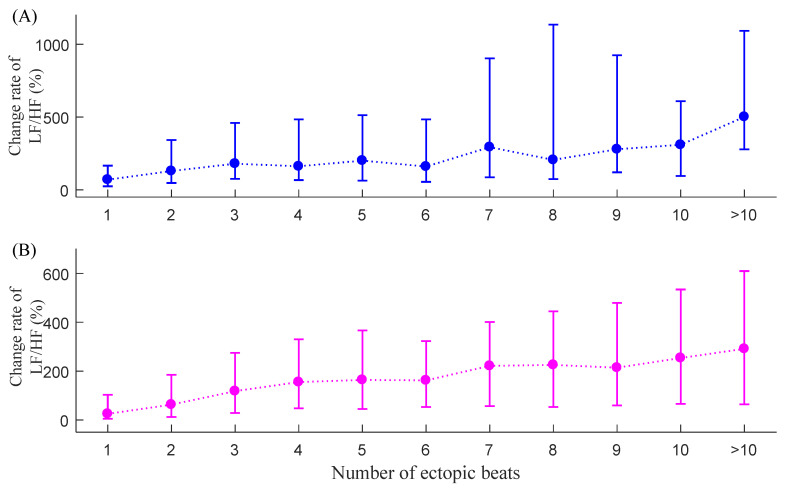
Trends of C_LF/HF_ under different ectopic beat burdens, i.e., the number of ectopic beats increase from 1 to more than 10: (**A**) NSR group and (**B**) CHF group.

Figure 8 shows the corresponding median (IQR) results of C_LF/HF_ for the two groups. The change rate index C_LF/HF_ also increased with the increase of the number of ectopic beats, and the influence appeared to be more profound compared with that of SDNN. For NSR subjects, C_LF/HF_ results were 69.8% (24.5–166.2%) when only one ectopic beat was included in each 5 min RR segment and fluctuated to 501.2% (278.1–1091.7%) when more than 10 ectopic beats were included. For CHF patients, C_LF/HF_ results were 25.0% (4.7–102.9%) when only one ectopic beat was included and increased to 291.2% (64.0–609.4%) when more than 10 ectopic beats were included. The C_LF/HF_ results from the two groups indicated that the frequency-domain index of LF/HF was heavily influenced by ectopic beats, and the influence became heavier when the number of ectopic beats increased.

### 3.3. C_SampEn_

**Figure 9:**
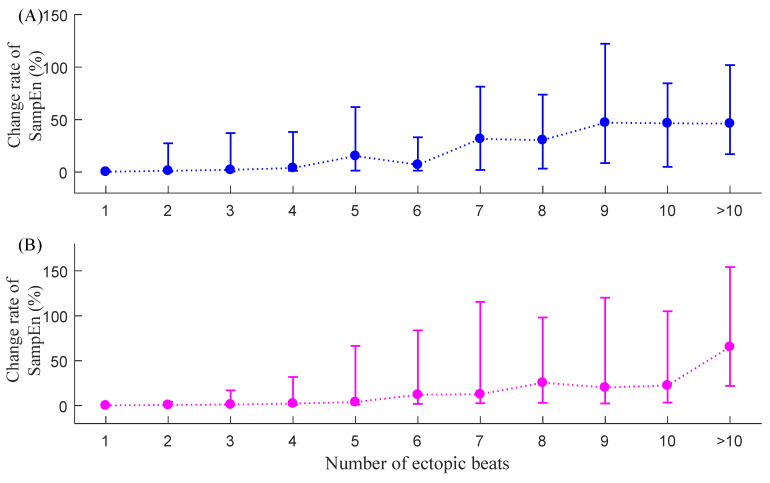
Trends of C_SampEn_ under different ectopic beat burdens, i.e., the number of ectopic beats increase from 1 to more than 10: (**A**) NSR group and (**B**) CHF group.

Figure 9 shows the corresponding median (IQR) results of C_SampEn_ for the two groups. The change rate index C_SampEn_ still increased with the increase in the number of ectopic beats. For NSR subjects, the C_SampEn_ results were 0.3% (0.1–1.6%) when only with one ectopic beat and became larger as the ectopic number increased, yielding a maximum value of 46.2% (17.1–101.8%) when the ectopic number was more than 10. For CHF patients, the C_SampEn_ results were 0.2% (0.04–0.7%) when there was only one ectopic beat and achieved maximum results of 65.4% (21.8–154.3%) when the ectopic number was more than 10. Variation of C_SampEn_ also had a general increasing trend related to the ectopic numbers, indicated by the increased IQR range with the increase in the number of ectopic beats. 

### 3.4. C_PT-SampEn_

**Figure 10:**
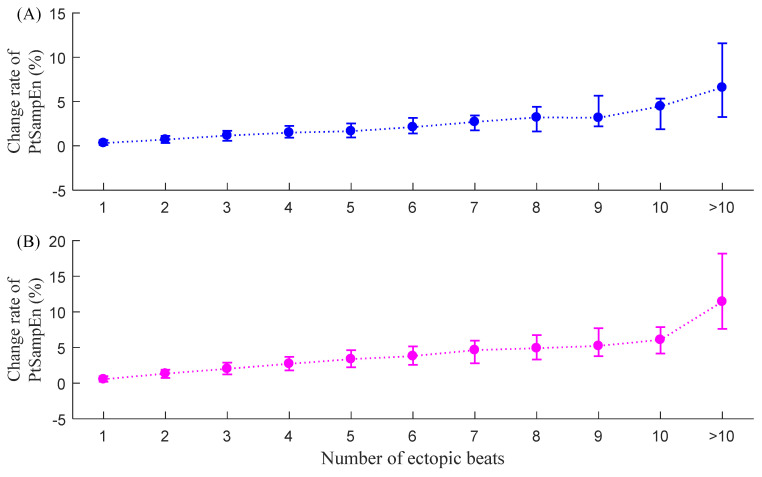
Trends of C_Pt-SampEn_ under different ectopic beat burdens, i.e., the number of ectopic beats increase from 1 to more than 10: (**A**) NSR group and (**B**) CHF group.

Figure 10 shows the corresponding median (IQR) results of the new entropy index C_Pt-SampEn_ for the two groups. C_Pt-SampEn_ shows significantly lower values compared with the other three indices. For NSR subjects, C_Pt-SampEn_ was only 0.3% (0.1–0.6%) when only one ectopic beat was included and increased to 6.6% (3.3–11.6%) when more than 10 ectopic beats were included. For CHF patients, C_Pt-SampEn_ was 0.6% (0.2–1.0%) when there was only one ectopic beat and increased to 11.4% (7.6–18.2%) when more than 10 ectopic beats were included. The C_Pt-SampEn_ results from the two groups indicated that the influence of ectopic beat burden on Pt-SampEn appeared to be relatively small. An obvious change in C_Pt-SampEn_ was only observed from the RR segments with lots of ectopic beats (>10 in this study).

### 3.5. Comparison of Variances of the Change Rate Index

Table 3 shows the variances of the four indices before and after ectopic beats removal in each ectopic number group. The HRV indices can increase or decrease after the ectopic beats were removed, and, herein, we focused on their changes (i.e., standard deviation (std)). From Table 3, we could see that, except for some small ups and downs, the std of all four change rate indices roughly increased with the increasing number of ectopic beats, and among the four indices, the std of C_LF/HF_ was the largest with 730.5% for NSR subjects and 388.0% for CHF patients, respectively. The std of C_SDNN_ and C_SampEn_ were also large and were 16.4% and 58.3% for NSR subjects, 20.3% and 74.9% for CHF patients. The std of C_Pt-SampEn_ was relatively small, only 2.3% for NSR subjects and 3.3% for CHF patients. These results further confirmed that, compared to other indices, Pt-SampEn had better robustness to ectopic beats in each ectopic number group and, thus, had better stability. 

## 4. Discussion and Conclusions

The analysis of HRV provides a useful tool for the evaluation of cardiovascular functions. There are generally three types of methods for HRV analyses: (1) time-domain analyses such as SDNN, RMSSD, SDANN, etc.; (2) frequency-domain analyses such as LF, HF, and LF/HF, etc.; (3) non-linear analyses such as the Lorentz model, Markov models, coarse-graining spectral analysis, and entropy measure analysis, etc. Ectopic beats are generated by additional electrical impulses imposed by other latent pacemakers; in former research, we found that ectopic beats might cause bias in HRV measurements, in time-domain, frequency-domain, and entropy measurement analysis [20].

In this study, the effects of different amounts of ectopic beats on four HRV indices were explored. The results showed that all four indices changed after the removal of ectopic beats, and the relative changes (change rate index C) also increased in general with the increase of the burden of ectopic beats. Among them, the frequency-domain index LF/HF showed the largest relative changes for both NSR and CHF groups, indicating that this index was most seriously affected by ectopic beats and followed by the indices of SDNN and SampEn. Index Pt-SampEn reported the least change rate index C results, indicating its better robustness and stability for dealing with the ectopic RR segments. 

Ectopic beats usually lead to sudden changes in RR intervals, and the results showed, as indicated above, that the HRV indices LF/HF, SDNN, and SampEn were much more sensitive to these sudden changes. However, Pt-SampEn had better robustness for ectopic beats than the former three indices. Compared to SampEn, Pt-SampEn used a physical meaning threshold [28]. The threshold *r* value was the most difficult parameter to be determined among all of the three intrinsic parameters [27]. Researchers developed various methods for *r* value selection [29,30], but it seems that different methods perform well only under certain circumstances. The concept of the physical threshold was partly from a study of AF detection use SampEn [31], and our previous study certified that this physical threshold could perform well on NSR and CHF RR interval time series with ectopic beats [25] since it could avoid invalid entropy values in each RR segment, and a more stable specific *r* value could be determined by the sampling the resolution of physiological signals. The reason could be explained by the entropy calculation process. As described in the Methods section, the raw ECGs were collected with a relatively low sample rate of 128 Hz, indicating that the minimum difference for any two RR intervals could be about 8 ms. As SampEn analyzed the Chebyshev distance between two RR interval time series, this distance would be an integral multiple of 8 ms, regardless the truncated error. If the threshold *r* was too small, i.e., smaller than 8 ms, similar vectors would be quite limited or even none, leading to invalid results in the SampEn calculation. Considering that the SD of a 5 min RR segment is usually dozens of ms, the traditionally recommended threshold *r* of 0.1 times SD of the signal had a high probability of being smaller than 8 ms, inducing invalid results in the SampEn analysis. Therefore, the physical threshold-based Pt-SampEn addressed this point and could avoid invalid entropy values. In addition, we observed the change of HRV indices during different burdens of ectopic beats. So it provided the possibility to use the HRV index as a predictor for the behavior of ectopic beats, which was ‘contrary thinking’ regarding the current study.

Reduced HRV or low HRV is usually regarded as an important prognostic factor for predicting sudden arrhythmic death after acute myocardial infarction, as well as for both arrhythmic and non-arrhythmic heart diseases, and even for mortality. Thus, there is no doubt that enough attention should be paid to accurately quantifying HRV. However, several limitations should be mentioned. First, we did not distinguish the non-stationary RR segments from the raw RR interval recordings. Magagnin et al. reported the potential bias of spectral, symbolic, and entropy HRV indices due to non-stationarities [32]. So there was a need to check the stationarity of 5 min RR segments and find ways to reject non-stationary segments, although the methods of detecting non-stationarities were challenging. Second, RR interval time series usually exhibit a non-linear characteristic, which necessitates the use of non-linear methods in order to reveal the different physiological or pathological situations in the heart rate. Thus, non-linear analysis methods, such as entropy measures, have been widely studied in the past 10–20 years. However, for special tests (the graded head-up tilt), the linear model-based approach for the estimation of conditional entropy was highly related to non-linear model-free (MF) techniques like ApEn and SampEn [33], indicating that we should re-recognize the reasonable applications of the existing entropy measures. Last, new entropy measures should also be deeply exploited. Porta et al. proposed a K-nearest-neighbor entropy method to estimate the conditional entropy by considering the control of the loss of reliability of the conditional distributions by pattern length without introducing a priori information, and showed good performance on the test of 250-sample RR interval time series [34]. The usefulness of this K-nearest-neighbor entropy on the ectopic RR segments should be further studied. We identified this point as our future work. 

In conclusion, ectopic beats might cause bias in different HRV measurement indices, and the affection trend increased when the ectopic number increased, but Pt-SampEn had better performance in time series with ectopic beats due to its physical threshold.

## Figures and Tables

**Table 1:** Data profile for the normal sinus rhythm (NSR) group from the PhysioNet/MIT RR Interval Database. # Number of.

Record	# Ectopic-Free 5 min Segments	# Ectopic 5 min Segments	Record	# Ectopic-Free 5 min Segments	# Ectopic 5 min Segments
NSR001	212	58	NSR028	190	95
NSR002	134	146	NSR029	269	18
NSR003	247	37	NSR030	229	58
NSR004	245	33	NSR031	97	191
NSR005	89	198	NSR032	99	188
NSR006	239	40	NSR033	261	14
NSR007	203	81	NSR034	265	18
NSR008	237	50	NSR035	258	29
NSR009	262	25	NSR036	254	28
NSR010	168	107	NSR037	246	29
NSR011	195	92	NSR038	251	4
NSR012	247	40	NSR039	200	87
NSR013	249	32	NSR040	266	17
NSR014	175	112	NSR041	253	29
NSR015	263	24	NSR042	277	10
NSR016	245	42	NSR043	159	123
NSR017	22	265	NSR044	17	270
NSR018	73	213	NSR045	135	149
NSR019	254	33	NSR046	181	94
NSR020	172	108	NSR047	266	22
NSR021	275	12	NSR048	268	21
NSR022	236	47	NSR049	285	3
NSR023	253	34	NSR050	285	3
NSR024	15	272	NSR051	281	6
NSR025	166	120	NSR052	274	10
NSR026	243	44	NSR053	269	1
NSR027	280	5	NSR054	271	8

**Table 2:** Data profile for the congestive heart failure (CHF) group from the PhysioNet/MIT RR Interval Database. # Number of.

Record	# Ectopic-Free 5 min Segments	# Ectopic 5 min Segments	Record	# Ectopic-Free 5 min Segments	# Ectopic 5 min Segments
CHF201	240	36	CHF216	250	14
CHF202	97	150	CHF217	53	228
CHF203	75	187	CHF218	47	217
CHF204	0	247	CHF219	236	28
CHF205	31	245	CHF220	138	143
CHF206	11	240	CHF221	0	276
CHF207	1	249	CHF222	1	274
CHF208	31	257	CHF223	0	274
CHF209	70	156	CHF224	137	150
CHF210	16	258	CHF225	97	121
CHF211	275	11	CHF226	18	257
CHF212	0	205	CHF227	0	275
CHF213	7	281	CHF228	71	204
CHF214	0	204	CHF229	267	20
CHF215	110	166			

**Table 3:** The standard deviation (std) of the change rate index of different HRV indices. # Number of.

# Ectopic Beat	Variances in NSR Subjects (%), std				Variances in CHF Patients (%), std			
	C_SDNN_	C_LF/HF_	C_SampEn_	C_Pt-SampEn_	C_SDNN_	C_LF/HF_	C_SampEn_	C_Pt-SampEn_
1	9.3	222.5	29.5	0.4	13.3	177.7	26.7	0.6
2	12.1	415.3	40.2	0.6	16.9	262.2	41.8	1.1
3	14.5	547.9	49.2	1.0	18.6	334.2	61.6	1.6
4	16.3	483.5	55.5	1.2	20.3	385.4	68.2	2.1
5	16.4	742.1	59.7	1.7	20.8	354.4	71.4	2.4
6	15.1	520.7	47.9	1.7	21.0	338.2	79.1	2.2
7	18.5	679.5	55.7	1.3	22.0	356.1	71.2	2.5
8	18.2	1001.8	65.9	1.9	21.0	375.3	91.2	2.8
9	21.1	818.6	77.0	2.4	22.0	571.1	86.5	2.7
10	19.3	1037.3	84.8	3.2	22.2	524.1	92.1	3.4
>10	19.7	1566.0	75.4	9.4	25.3	589.7	134.0	15.0
Mean	16.4	730.5	58.3	2.3	20.3	388.0	74.9	3.3

## Data Availability

The Data included in this study are all from the open-access PhysioNet database and will not be transmitted.
